# Susceptibility of bacterial endophthalmitis isolates to vancomycin, ceftazidime, and amikacin

**DOI:** 10.1038/s41598-021-95458-w

**Published:** 2021-08-05

**Authors:** Kuan-Jen Chen, Ming-Hui Sun, Chiun-Ho Hou, Hung-Chi Chen, Yen-Po Chen, Nan-Kai Wang, Laura Liu, Wei-Chi Wu, Hung-Da Chou, Eugene Yu-Chuan Kang, Chi-Chun Lai

**Affiliations:** 1 Department of Ophthalmology, Chang Gung Memorial Hospital, Taoyüan, Taiwan; 2 College of Medicine, Chang Gung University, Taoyüan, Taiwan; 3 Department of Ophthalmology, Tucheng Municipal Hospital, New Taipei, Taiwan; 4 Department of Ophthalmology, Edward S. Harkness Eye Institute, Columbia University, New York, NY USA; 5 Department of Ophthalmology, Chang Gung Memorial Hospital, Keelung, Taiwan

**Keywords:** Diseases, Medical research, Antimicrobials

## Abstract

Bacterial endophthalmitis is a rare intraocular infection, and prompt administration of intravitreal antibiotics is crucial for preventing severe vision loss. The retrospective study is to investigate the in vitro susceptibility to the antibiotics vancomycin, amikacin, and ceftazidime of bacterial endophthalmitis isolates in specimens at a tertiary referral center from January 1996 to April 2019 in Taiwan. Overall, 450 (49.9%) isolates were Gram positive, 447 (49.6%) were Gram negative, and 4 (0.4%) were Gram variable. In Gram-positive isolates, coagulase-negative staphylococci were the most commonly cultured bacteria (158, 35.1%), followed by *Streptococci* (100, 22.2%), *Enterococci* (75, 16.7%), and *Staphylococcus aureus* (70, 15.6%). In Gram-negative isolates, they were *Klebsiella pneumoniae* (166, 37.1%) and *Pseudomonas aeruginosa* (131, 29.3%). All Gram-positive organisms were susceptible to vancomycin, with the exception of one *Enterococcus faecium* isolate (1/450, 0.2%). Of the Gram-negative isolates, 96.9% and 93.7% were susceptible to ceftazidime and amikacin, respectively. Nine isolates (9/447, 2.0%) were multidrug-resistant Gram-negative bacteria, comprising *K. pneumoniae* (4/164, 2.4%), *Acinetobacter baumannii* (2/3, 67%), and *Stenotrophomonas maltophilia* (3/18, 17%). In conclusion, in vitro susceptibility testing revealed that vancomycin remains the suitable antibiotic treatment for Gram-positive endophthalmitis. Ceftazidime and amikacin provide approximately the same degree of Gram-negative coverage. Multidrug-resistant bacterial endophthalmitis was uncommon.

## Introduction

Bacterial endophthalmitis is a rare intraocular infection that can occur following ocular surgery or trauma, as well as through the hematogenous spread of microorganisms from endogenous infection. Prompt administration of intravitreal antibiotics with or without pars plana vitrectomy is crucial for preventing severe vision loss. Both the bacterial spectrum and antibiotic susceptibility patterns in bacterial endophthalmitis isolates must be considered in the context of treatment. Vancomycin is a first-line drug for managing Gram-positive bacterial endophthalmitis, whereas ceftazidime or amikacin are typically used for Gram-negative coverage. Amid growing concern over the emergence of multidrug-resistant (MDR) bacteria, selecting antibiotics for infection treatment has become a critical issue. Common MDR organisms include (1) vancomycin-resistant enterococci; (2) methicillin-resistant *S. aureus*; (3) extended-spectrum β-lactamase (ESBL)-producing Gram-negative bacteria; (4) carbapenemase-producing *K. pneumoniae*; and (5) MDR Gram-negative bacteria, such as *K. pneumoniae*, *Enterobacter* spp., *Escherichia coli*, *A. baumannii*, *P. aeruginosa*, and *S. maltophilia.* This group of Gram-positive and Gram-negative bacteria has been named the ESKAPE group (*E. faecium*, *S. aureus*, *K. pneumoniae*, *A. baumannii*, *P. aeruginosa*, and *Enterobacter* spp.)^1^. Studies have been conducted on MDR bacterial endophthalmitis^2–8^. Although most isolates causing ocular infection are not caused by MDR bacteria, antibiotic susceptibility testing may reveal trends in antimicrobial susceptibility and resistance.

This study investigated the in vitro susceptibility of Gram-positive and Gram-negative bacterial endophthalmitis isolates collected over 23 years to vancomycin, ceftazidime, and amikacin. MDR pathogens were also examined.

## Methods

The protocol of this retrospective, noncomparative laboratory case series was approved by the Institutional Review Board (IRB number: 201900614B0C601, 10 Aug 2019) of Chang Gung Memorial Hospital in Taiwan, and the requirement for written informed consent from the patients from whom the specimens were collected was waived. All clinical procedures were conducted according to the principles of the Declaration of Helsinki. The microbiological data of bacterial cultures isolated from culture-confirmed bacterial endophthalmitis at the Department of Microbiology of the participating hospital between January 1996 and April 2019 were reviewed. To protect the patients’ privacy, the data were deidentified. Data on clinical presentations and visual outcomes were not part of the laboratory records.

The bacterial isolates are from intraocular samples (anterior chamber, vitreous, and/or vitrectomy specimens). All microbiology investigations were performed at Microbiology Department, Chang Gung Memorial Hospital, Taoyuan, Taiwan. Bacterial culture isolates were identified by conventional microbiological methods (January 1996 to December 2013) and matrix-assisted laser desorption/ionization time of flight mass spectrometry (MALDI-TOF-MS) (January 2014 to April 2019). Conventional microbiological methods included Gram-staining and biochemical tests. In MALDI-TOF-MS, automatic measurement of the spectrum and comparative analysis with reference spectra of bacteria were performed using an Ultraflextreme mass spectrometer and MALDI-Biotyper 3.0 software (Bruker Daltonics). The reliability of identification in the MALDI Biotyper system was expressed in points. A log(score) ≥ 2.0 indicated identification to the species level. The isolates were tested for susceptibility to various antibiotics using the Kirby Bauer Disk diffusion method on Mueller Hinton blood agar. The Clinical and Laboratory Standards Institute (Wayne, PA) standards were used for interpretation and quality control for each corresponding year^9^. In vitro susceptibility of cultured bacterial organisms, the tested antibiotics mostly included either vancomycin for Gram-positive bacteria or ceftazidime and amikacin for Gram-negative bacteria. All data from the antibiotic susceptibility testing were further reviewed in the Gram-positive bacterial isolates resistant to vancomycin and in the Gram-negative bacteria resistant to either ceftazidime or amikacin. To be considered MDR, organisms must be resistant to antimicrobial drugs of three or more classes. Although methicillin-resistant *S. aureus* (MRSA) was susceptible to vancomycin, MRSA was not defined as an MDR organism in this study.

## Results

A total of 901 bacterial endophthalmitis isolates were cultured over the 23-year study period. An overview of the isolates is provided in Table 1.

**Table 1:** Bacteria isolated from patients diagnosed with endophthalmitis.

Gram-positive	Genus or species	450	49.9%
Cocci	*Staphylococcus* spp.		
	CoNS	158	17.5%
	*S. aureus*	70	7.8%
	*Streptococcus* spp.	100	11.1%
	*Enterococcus* spp.	75	8.3%
	*Aerococcus* spp.	1	0.1%
	*Micrococcus* spp.	3	0.3%
	*Rothia mucilaginosa*	1	0.1%
Bacilli	*Corynebacterium* spp.	9	1.0%
	*Cutibacterium* acnes	10	1.1%
	*Paenibacillus* spp.	1	0.1%
	*Bacillus* spp.	18	2.0%
	*Clostridium* spp.	4	0.4%

Gram-negative	Genus or species	447	49.6%
Cocci	*Neisseria* spp.	4	0.4%
Coccobacilli	*Hemophilus* spp.	11	1.2%
**Bacilli**			
*Enterobacteriaceae*	*Klebsiella* spp.	166	18.4%
	*Citrobacter* spp.	8	0.9%
	*Enterobacter* spp.	16	1.8%
	*Morganella* spp.	2	0.2%
	*Proteus* spp.	12	1.3%
	*Salmonella* spp.	2	0.2%
	*Serratia* spp.	12	1.3%
	*Escherichia* spp.	13	1.4%
*Non-Enterobacteriaceae-*fermentative	*Aeromonas* spp.	3	0.3%
	*Vibrio* spp.	1	0.1%
*Non-Enterobacteriaceae-*nonfermentative	*Acinetobacter* spp.	5	0.6%
	*Burkholderia* spp.	4	0.4%
	*Flavobacterium* spp.	3	0.3%
	*Moraxella* spp.	4	0.4%
	*Pseudomonas* spp.	131	14.5%
	*Roseomonas* spp.	1	0.1%
	*Stenotrophomonas maltophilia*	18	2.0%
	Others NF-GNB	29	3.2%
Anaerobic	*Bacteroides* spp.	1	0.1%
	*Prevotella* spp.	1	0.1%

Gram variable bacilli	Genus or species	4	0.4%
Nontuberculous	*Mycobacterium chelonae*	2	0.2%
*Mycobacteria*	*Mycobacterium abscesses*	1	0.1%
*Nocardia* spp.		1	0.1%
Total		901	100%

*CoNS* coagulase-negative Staphylococcus, *NF-GNB* non-fermentative-Gram-negative bacilli.

### Organismal spectrum

Overall, 450 (49.9%) of isolates were Gram positive, 447 (49.6%) were Gram negative, and 4 (0.4%) were Gram variable. In the 450 Gram-positive isolates, coagulase-negative staphylococci (CoNS) were the most commonly cultured bacterial organisms (158, 35.1%), followed by streptococci (100, 22.2%), enterococci (75, 16.7%), and *S. aureus* (70, 15.6%). In the 447 Gram-negative isolates, they were *K. pneumoniae* (166, 37.1%) and *P. aeruginosa* (131, 29.3%).

### Susceptibility of Gram-positive isolates to vancomycin

Table 2 presents the susceptibility of the Gram-positive isolates to vancomycin. All Gram-positive bacteria, with the exception of one *E. faecium* isolate, were susceptible to vancomycin, including CoNS, *S. aureus*, *Enterococcus faecalis*, and streptococci (Table 2).

**Table 2:** Vancomycin susceptibility testing in Gram positive bacteria.

Gram-positive bacteria		Vancomycin	
		Number	%
***Staphylococcus* spp.**			
CoNS	158	158/158	100%
*S. aureus*	70	70/70	100%
*Streptococcus* spp.	100	100/100	100%
*Enterococcus* spp.	75	74/75	98.7%
*Aerococcus* spp.	1	1/1	100%
*Micrococcus* spp.	3	3/3	100%
*Rothia mucilaginosa*	1	1/1	100%
*Corynebacterium* spp.	9	9/9	100%
*Cutibacterium* acnes	10	10/10	100%
*Paenibacillus* spp.	1	1/1	100%
*Bacillus* spp.	18	18/18	100%
*Clostridium* spp.	4	4/4	100%
	450	449/450	99.8%

*CoNS* coagulase-negative *Staphylococcus.*

### Susceptibility of Gram-negative isolates to amikacin and ceftazidime

Table 3 presents the susceptibility of the Gram-negative isolates to amikacin and ceftazidime. Overall, 96.9% (413/426) were susceptible to ceftazidime and 93.7% (401/428) were susceptible to amikacin. Regarding the most commonly isolated Gram-negative organisms, 98% of the *K. pneumoniae* isolates were susceptible to both ceftazidime and amikacin. Of the *P. aeruginosa* isolates, 98% and 95% were susceptible to ceftazidime and amikacin, respectively. Of the *S. maltophilia* isolates, 8% and 46% were susceptible to ceftazidime and amikacin, respectively. All the *Haemophilus influenzae*, *Serratia marcescens*, *Enterobacter* spp., *Citrobacter* spp., and *E. coli* isolates were susceptible to ceftazidime and amikacin. Among them, MDR Gram-negative bacteria comprised *K. pneumoniae* (4/164, 2.4%), *A. baumannii* (2/3, 67%), and *S. maltophilia* (3/18, 17%). The *S. maltophilia* isolates exhibited high resistance to ceftazidime (11/12, 94%) and amikacin (7/13, 54%).

**Table 3:** Susceptibility of Gram-negative bacterial isolates to ceftazidime and amikacin.

Gram-negative	Genus	Species	No.	Amikacin	Ceftazidime
Cocci	*Neisseria*		4	1/1 (100%)	1/1 (100%)
Coccobacilli	*Hemophilus*	*H. influenzae*	11	11/11 (100%)	11/11 (100%)
**Bacilli**					
*Enterobacteriaceae*	*Klebsiella*	*K. pneumoniae*	164	161/164 (98%)	160/164 (98%)
		*K. oxytoca*	2	2/2 (100%)	2/2 (100%)
	*Citrobacter*	*C. freundii*	4	4/4 (100%)	4/4 (100%)
		*C. koseri*	4	4/4 (100%)	4/4 (100%)
	*Enterobacter*	*E. aerogenes*	1	1/1 (100%)	1/1 (100%)
		*E. agglomerans*	2	2/2 (100%)	2/2 (100%)
		*E. cloacae*	11	11/11 (100%)	11/11 (100%)
		*E. gergoviae*	2	2/2 (100%)	2/2 (100%)
	*Morganella*	*M. morganii*	2	2/2 (100%)	2/2 (100%)
	*Proteus*	*P. mirabilis*	11	11/11 (100%)	11/11 (100%)
		*P. vulgaris*	1	1/1 (100%)	1/1 (100%)
	*Salmonella*		2	NT	2/2 (100%)
	*Serratia*	*S. marcescens*	12	12/12 (100%)	12/12 (100%)
	*Escherichia*	*E. coli*	13	13/13 (100%)	13/13 (100%)
*Non-Enterobacteriaceae-fermentative*	*Aeromonas*	*A. hydrophilia*	3	3/3 (100%)	3/3 (100%)
	*Vibrio*	*V. parahaemolyticus*	1	NT	1/1 (100%)
*Non-Enterobacteriaceae-nonfermentative*	*Acinetobacter*	*A. baumannii*	3	1/3 (33%)	1/3 (33%)
		*A. lwoffii*	1	1/1 (100%)	1/1 (100%)
		*A. pittii*	1	1/1 (100%)	1/1 (100%)
	*Burkholderia*	*B. pseudomallei*	1	0/1 (0%)	1/1 (100%)
		*B. cepacia complex*	3	0/1 (0%)	3/3 (100%)
	*Flavobacterium*		3	3/3 (100%)	3/3 (100%)
	*Moraxella*	*M. catarrhalis*	3	NT	3/3 (100%)
		*M. nonliquefaciens*	1	NT	1/1 (100%)
	*Pseudomonas*	*P. aeruginosa*	123	117/123 (95%)	121/123 (98%)
		*P. fluorescens*	2	2/2 (100%)	2/2 (100%)
		*P. luteola*	1	1/1 (100%)	1/1 (100%)
		*P. stutzeri*	2	2/2 (100%)	2/2 (100%)
		*Non-identified*	3	NT	NT
	*Roseomonas*		1	1/1 (100%)	0/1 (0%)
	*Stenotrophomonas*	*S. maltophilia*	18	6/13 (46%)	1/12 (8%)
	Others NF-GNB		29	22/29 (76%)	25/29 (86%)
Anaerobic	*Bacteroides*		1	NT	NT
	*Prevotella*		1	NT	NT
			447	401/428 (93.7%)	413/426 (96.9%)

*NFGNB* nonfermenting Gram-negative bacilli, *NT* not tested.

### Susceptibility of Gram-variable isolates

Two *Mycobacterium chelonae*, one *Mycobacterium abscessus*, and one *Nocardia* spp. isolate comprised the Gram-variable isolates. Three *Mycobacterium* isolates were susceptible to amikacin but were not tested for susceptibility to ceftazidime. The *Nocardia* spp. isolate was not tested for susceptibility to amikacin and ceftazidime.

### MDR organisms

Ten isolates (10/901, 1.1%), including one *E. faecium*, four *K. pneumoniae*, two *A. baumannii*, and three *S. maltophilia*, were MDR organisms. Table 4 shows the antibiotic susceptibility testing results for multidrug resistance. The *E. faecium* isolate was susceptible to linezolid and tigecycline but resistant to teicoplanin. ESBL production was detected in the four *K. pneumoniae* isolates, of which only one was susceptible to amikacin. All four isolates were susceptible to carbapenems (imipenem, meropenem, or ertapenem). One of the *A. baumannii* isolates was susceptible to imipenem. The other was resistant to meropenem but susceptible to colistin and tigecycline. All three MDR *S. maltophilia* isolates were susceptible to amikacin but resistant to ceftazidime, trimethoprim-sulfamethoxazole, and fluoroquinolones, including levofloxacin and moxifloxacin.

**Table 4:** Antibiotic susceptibility testing in multidrug-resistant organisms.

Antibiotics	Organisms									
	*Enterococcus faecium*	*Klebsiella pneumoniae*				*Acinetobacter baumannii*		*Stenotrophomonas maltophilia*		
No. of isolates	1	1	2	3	4	1	2	1	2	3
Penicillin	R									
Ampicillin	R	R	R	R		R				
Amoxicillin-CLA					S					
Ampicillin-SUL							R			
Piperacillin		R	R	S	R	R	R			
Piperacillin-TZP			S		S		R			
Vancomycin	R									
Teicoplanin	R									
Linezolid	S									
Tigecycline	S						S	R	R	I
Gentamicin		R	R	R	R	R	R			
High level GM	R									
Amikacin		R	R	R	S	R	R	S	S	S
Cefazolin		R	R	R	R	R				
Cefuroxime		R	R	R	R	R		R	R	R
Ceftriaxone		R	R	R	R	R	R			
Ceftazidime		R	R	R	R	R	R	R	R	R
Floxomef		R	R	S	S	R				
Cefepime							R			
Aztreonam		R	R	R	R	R				
TMP-SMX		R	R	R		R		R	R	R
Ciprofloxacin		S	R	S	S	R	R			
Levofloxacin								R	R	R
Moxifloxacin								R	R	R
Imipenem		S		S		S				
Meropenem			S				R			
Ertapenem					S					
Colistin							S			

*CLA* clavulanic acid, *I* intermediate, *S* susceptible, *SUL* sulbactam, *R* resistant, *TMP-SXT* trimethoprim-sulfamethoxazole, *TZP* tazobactam.

## Discussion

Understanding of the various organisms causing bacterial endophthalmitis is critical to the development of effective treatments. Case studies on bacterial endophthalmitis have reported variability in the microbiological spectrum of causative pathogens according to the types of endophthalmitis and to the regions and countries where the studies were conducted. Bacterial endophthalmitis is caused by Gram-positive and Gram-negative organisms, as well as some Gram-variable organisms. Empirically supported antibiotic treatments for infectious endophthalmitis are developed on the basis of likely causative organisms and their susceptibility patterns. In this study, the proportions of Gram-positive and Gram-negative bacteria (49.9% and 49.6%, respectively) were approximately equal, with Gram-variable bacteria accounting for a considerably lower proportion (0.4%). Exogenous bacterial endophthalmitis mostly arises form Gram-positives because Gram-negatives are not common on the eyelid margins. This is partially attributable to the large number of reported cases of endogenous *K. pneumoniae* endophthalmitis and *P. aeruginosa* keratitis-related endophthalmitis in Taiwan^4,6,10–12^. Our data differ from those of other published reviews of endophthalmitis isolates (Table 5)^13–19^. Gram-positive bacteria account for most bacterial isolates of endophthalmitis in studies conducted in the United States, Canada, and Austria, whereas Gram-negative bacteria are predominant in India^13–19^. The higher proportion of Gram-negative isolates observed in one study^17^ from India may be explained by trauma and environmental factors. In this study, 1.1% (10/901) of the MDR bacterial isolates included one Gram-positive and nine Gram-negative bacterial organisms.

**Table 5:** Review and comparison of bacterial endophthalmitis isolates.

	UPMC^19^	NYEEI^13^	BPEI^15^	Toronto^18^	Hyderabad^17^	Hyderabad^14^	Queensland^16^	CGMH*
	USA	USA	USA	Canada	India	India	Australia	Taiwan
Study period	1993–2015	1987–2011	2002–2011	2000–2009	1991–2015	2010–2013	1998–2013	1996–2019
No. of isolates	665	943	375	265	2840	196	193	901
Gram-positive isolates	92.9%	89.2%	87.2%	90.2%	68.7%	37.2%	84.5%	49.9%
Susceptibility to vancomycin	99.7%	99.7%	100.0%	99.6%	96%	100.0%	100.0%	99.8%
Gram-negative isolates	7.1%	10.8%	12.8%	9.8%	32.3%	62.8%	15.5%	49.6%
Susceptibility to amikacin	95.7%	92.9%	N/A	N/A	64–67%^a^	87.0%	100.0%	93.7%
Susceptibility to ceftazidime	93.6%	91.5%	100.0%	100.0%	69–38%^a^	82.0%	100.0%	96.9%

*BPEI* Bascom Palmer Eye Institute, *CGMH* Chang Gung Memorial Hospital, *N/A* not available, *NYEEI* New York Eye and Ear Infirmary, *UPMC* University of Pittsburgh Medical Center.

*Including 4 Gram-variable isolates.

^a^2005 → 2015.

As mentioned, the most commonly cultured Gram-positive isolates were CoNS, followed by *Streptococcus* spp., *Enterococcus* spp., and *S. aureus*. These results are consistent with those from previous studies^13–17^. However, a higher proportion of *Enterococcus* spp., especially *E. faecalis*, was noted in this study, and it resulted from more *E. faecalis* endophthalmitis cases in Taiwan^20,21^*.* Gram-positive vancomycin-resistant endophthalmitis has become a critical clinical issue worldwide; this is indicated by the fact that *Enterococcus* spp. and *Staphylococcus* spp. were the most commonly reported organisms^7^. The rate of Gram-positive vancomycin resistance in the present study was 0.2%. All *Staphylococcus* spp. isolates were susceptible to vancomycin, but 1 of the 75 (1.3%) *Enterococcus* isolates, *E. faecium*, was resistant to vancomycin. Several case reports have examined vancomycin-resistant enterococcal endophthalmitis^22–24^. *E. faecalis* was less commonly resistant to vancomycin. In other case reports of endophthalmitis, *E. casseliflavus*^25^ and *E. faecium*^2^ exhibited higher rates of resistance to vancomycin*.* In a literature review^7^ of 27 types of vancomycin-resistant Gram-positive bacteria, *Enterococcus* spp., CoNS, *S. aureus*, and *Bacillus* spp. were the most common organisms.

In this study, 3.1% and 6.3% of the Gram-negative bacteria was resistant to ceftazidime and amikacin, respectively. In these resistant isolates, most Gram-negative bacteria were resistant to either ceftazidime or amikacin. However, five isolates (5/447, 1.1%) were resistant to both ceftazidime and amikacin. Apart from four *K. pneumoniae* isolates, most bacteria in the Enterobacteriaces family were susceptible to amikacin and ceftazidime. By comparison, nonfermenting Gram-negative bacteria not belonging to the Enterobacteriaces family were more likely to be resistant to ceftazidime and amikacin. *P. aeruginosa* had a comparable degree of susceptibility to ceftazidime (98%) and amikacin (95%). By contrast, the susceptibility of *S. maltophilia* to ceftazidime and amikacin differed substantially (8% and 46%, respectively).

Among the MDR bacteria, *E. faecium* was susceptible to linezolid and tigecycline. The nine MDR Gram-negative bacterial isolates comprised *K. pneumoniae* (4/164, 2.4%), *A. baumannii* (2/3, 67%), and *S. maltophilia* (3/18, 17%). The ceftazidime resistance of *K. pneumoniae*, a member of the Enterobacteriaceae family, was indicated by the presence of ESBL production. The ESBL-producing Gram-negative bacteria were resistant to all third-generation cephalosporins. Four MDR *K. pneumoniae* isolates were susceptible to carbapenems (imipenem, meropenem, or ertapenem). One of the *A. baumannii* isolates was susceptible to carbapenem, and the other was susceptible to colistin and tigecycline. All three MDR *S. maltophilia* isolates were susceptible to amikacin but resistant to ceftazidime, trimethoprim-sulfamethoxazole, and fluoroquinolones, including levofloxacin and moxifloxacin. In a case report of carbapenemase-producing *K. pneumoniae* endophthalmitis by Zhou et al.^8^, the *K. pneumoniae* isolate was resistant to amikacin, ceftazidime, and carbapenems but susceptible to polymyxin E and tigecycline. Roy et al.^26^ noted that four (100%) *A. baumannii* isolates were resistant to ceftazidime and one (25%) was resistant to amikacin. All four of those *A. baumannii* isolates were susceptible to ciprofloxacin^26^. Although *S. maltophilia* was typically susceptible to trimethoprim-sulfamethoxazole, levofloxacin, and moxifloxacin, Ji et al.^5^ reported on 14 patients with MDR *S. maltophilia* postcataract endophthalmitis. All eight *S. maltophilia* isolates were resistant to amikacin, imipenem, and ciprofloxacin but not to levofloxacin^5^. Five isolates were resistant to ceftazidime^5^.

Most bacteria are typically susceptible to vancomycin, ceftazidime, and amikacin at a concentration of ≤ 10 μg/mL. Interpretive standards for dilution susceptibility testing from the US Clinical and Laboratory Standards Institute indicate that the minimal inhibitory concentrations of vancomycin, ceftazidime, and amikacin are ≤ 4, ≤ 8, and ≤ 6 μg/mL, respectively^27^. The resistance concentrations of vancomycin, ceftazidime, and amikacin are ≥ 32, ≥ 32, and ≥ 64 μg/mL, respectively^27^. Intravitreal antibiotic injection is the principal treatment for infectious endophthalmitis. The susceptibility standards are based on serum standards used for systemic therapy from the Clinical and Laboratory Standards Institute. However, there are no standards for intravitreal therapy, and resistance is probably over-reported using the serum standards. In vitro resistance patterns may not be identical with in vivo susceptibility, and routinely administered intravitreal antibiotics typically deliver intraocular antibiotic concentrations that are considerably higher than the minimal inhibitory concentrations of most organisms. When patients’ conditions do not stabilize or improve after initial intravitreal antibiotic injection, additional injections of alternative antibiotics should be considered according to results in vitro susceptibility testing.

This study has some limitations. First, this was a single-center retrospective case series. Second, some Gram-negative isolates were not routinely tested at our hospital for susceptibility to amikacin and ceftazidime. Third, we did not retrieve accurate, detailed information on various types of endophthalmitis and visual outcomes, which may interest readers. Fourth, we did not analyze trends in the patterns of antibiotic susceptibility given the relatively small number of annual cases. Finally, we did not further analyze the MDR bacteria for specific resistance genes. Nevertheless, our findings contribute to the literature in providing a thorough analysis of the antimicrobial susceptibility of bacterial isolates from all patients diagnosed as having bacterial endophthalmitis over 23 years at a tertiary medical center.

In conclusion, the most commonly detected organisms in the Gram-positive and Gram-negative bacteria were CoNS and *K. pneumoniae*, respectively. MDR bacterial endophthalmitis was not common. Vancomycin remains the antibiotic of choice for the treatment of Gram-positive endophthalmitis. Amikacin and ceftazidime appear to provide approximately equal Gram-negative coverage.

## References

[1] Boucher HW (2009). Bad bugs, no drugs: No ESKAPE! An update from the Infectious Diseases Society of America. Clin. Infect. Dis. Off. Publ. Infect. Dis. Soc. Am..

[2] Bains HS, Weinberg DV, Feder RS, Noskin GA (2007). Postoperative vancomycin-resistant *Enterococcus faecium* endophthalmitis. Arch. Ophthalmol. (Chicago Ill. 1960).

[3] Chen KJ (2008). Endophthalmitis caused by *Acinetobacter baumannii*: Report of two cases. J. Clin. Microbiol..

[4] Chen KJ (2009). Endogenous Klebsiella endophthalmitis associated with *Klebsiella pneumoniae* pneumonia. Ocul. Immunol. Inflamm..

[5] Ji Y (2015). Post-cataract endophthalmitis caused by multidrug-resistant *Stenotrophomonas maltophilia*: Clinical features and risk factors. BMC Ophthalmol..

[6] Li YH (2018). Infectious sources, prognostic factors, and visual outcomes of endogenous *Klebsiella pneumoniae* endophthalmitis. Ophthalmol. Retina.

[7] Relhan N (2016). Endophthalmitis caused by Gram-positive organisms with reduced vancomycin susceptibility: Literature review and options for treatment. Br. J. Ophthalmol..

[8] Zhou Y, Wang X, Shen J, Lu Z, Liu Y (2019). Endogenous Endophthalmitis caused by Carbapenem-resistant Hypervirulent *Klebsiella Pneumoniae*: A case report and literature review. Ocul. Immunol. Inflamm..

[9] Clinical and Laboratory Standards Institute. *Performance Standards for Antimicrobial Disk Susceptibility Tests; Approved Standard*, 12th edn. M02-A13. (Clinical and Laboratory Standards Institute).

[10] Chen YH (2019). Prognostic factors and visual outcomes of pyogenic liver abscess-related endogenous *Klebsiella pneumoniae* endophthalmitis: A 20-year retrospective review. Sci. Rep..

[11] Chen KJ (2011). Endophthalmitis caused by *Pseudomonas aeruginosa* in Taiwan. Retina (Phila. Pa.).

[12] Chen KJ, Wu WC, Sun MH, Lai CC, Chen TL (2010). Pseudomonas endophthalmitis. Ophthalmology.

[13] Gentile RC (2014). Microbiological spectrum and antibiotic sensitivity in endophthalmitis: A 25-year review. Ophthalmology.

[14] Reddy AK (2015). Susceptibility of bacterial isolates to vancomycin and ceftazidime from patients with endophthalmitis: Is there a need to change the empirical therapy in suspected bacterial endophthalmitis?. Int. Ophthalmol..

[15] Schimel AM, Miller D, Flynn HW (2013). Endophthalmitis isolates and antibiotic susceptibilities: A 10-year review of culture-proven cases. Am. J. Ophthalmol..

[16] Moloney TP, Park J (2014). Microbiological isolates and antibiotic sensitivities in culture-proven endophthalmitis: A 15-year review. Br. J. Ophthalmol..

[17] Joseph J (2019). Trends in microbiological spectrum of endophthalmitis at a single tertiary care ophthalmic hospital in India: A review of 25 years. Eye (Lond.).

[18] Assaad D (2015). Bacterial endophthalmitis: 10-year review of the culture and sensitivity patterns of bacterial isolates. Can. J. Ophthalmol. J. Can. D'ophtalmol..

[19] Kodati S, Eller AW, Kowalski RP (2017). The susceptibility of bacterial endophthalmitis isolates to vancomycin, ceftazidime, and amikacin: A 23 year-review. Ophthalmol. Retina.

[20] Chen KJ (2009). Postcataract endophthalmitis caused by *Enterococcus faecalis*. Ocul. Immunol. Inflamm..

[21] Chen KJ (2021). *Enterococcus faecalis* endophthalmitis: Clinical settings, antibiotic susceptibility, and management outcomes. Microorganisms.

[22] Dave VP (2020). Enterococcus endophthalmitis: Clinical settings, antimicrobial susceptibility, and management outcomes. Retina (Phila. Pa.).

[23] Rishi E, Rishi P, Nandi K, Shroff D, Therese KL (2009). Endophthalmitis caused by *Enterococcus faecalis*: A case series. Retina (Phila. Pa.).

[24] Tang CW, Cheng CK, Lee TS (2007). Community-acquired bleb-related endophthalmitis caused by vancomycin-resistant enterococci. Can. J. Ophthalmol. J. Can. D'ophtalmol..

[25] Low JR, Teoh CS, Chien JM, Huang EH (2015). *Enterococcus casseliflavus* endophthalmitis due to metallic intraocular foreign body. Eye (Lond.).

[26] Roy R (2013). Endophthalmitis caused by *Acinetobacter baumanni*: A case series. Eye (Lond.).

[27] Performance Standards for Antimicrobial Susceptibility Testing. Clinical and Laboratory Standards Institute (CLSI). M100 28th edition (2018).

